# Retinal organoids provide unique insights into molecular signatures of inherited retinal disease throughout retinogenesis

**DOI:** 10.1111/joa.13768

**Published:** 2022-09-29

**Authors:** Avril Watson, Majlinda Lako

**Affiliations:** ^1^ Biosciences Institute Newcastle University Newcastle upon Tyne UK

**Keywords:** disease modelling, inherited retinal disease, retinal organoids, retinitis pigmentosa, retinoblastoma, retinal pigment epithelium, Stargardt disease, stem cells

## Abstract

The demand for induced pluripotent stem cells (iPSC)‐derived retinal organoid and retinal pigment epithelium (RPE) models for the modelling of inherited retinopathies has increased significantly in the last decade. These models are comparable with foetal retinas up until the later stages of retinogenesis, expressing all of the key neuronal markers necessary for retinal function. These models have proven to be invaluable in the understanding of retinogenesis, particular in the context of patient‐specific diseases. Inherited retinopathies are infamously described as clinically and phenotypically heterogeneous, such that developing gene/mutation‐specific animal models in each instance of retinal disease is not financially or ethically feasible. Further to this, many animal models are insufficient in the study of disease pathogenesis due to anatomical differences and failure to recapitulate human disease phenotypes. In contrast, iPSC‐derived retinal models provide a high throughput platform which is physiologically relevant for studying human health and disease. They also serve as a platform for drug screening, gene therapy approaches and in vitro toxicology of novel therapeutics in pre‐clinical studies. One unique characteristic of stem cell‐derived retinal models is the ability to mimic in vivo retinogenesis, providing unparalleled insights into the effects of pathogenic mutations in cells of the developing retina, in a highly accessible way. This review aims to give the reader an overview of iPSC‐derived retinal organoids and/or RPE in the context of disease modelling of several inherited retinopathies including Retinitis Pigmentosa, Stargardt disease and Retinoblastoma. We describe the ability of each model to recapitulate in vivo disease phenotypes, validate previous findings from animal models and identify novel pathomechanisms that underpin individual IRDs.

## Graphical abstract

Retinal organoids have become an exceedingly useful tool in recent years for understanding retinogenesis, particularly in the context of retinal disease. Pluripotent stem cells (PSCs) can be derived directly from somatic cells of affected patients, or alternatively, existing pluripotent lines can undergo gene editing to harbour mutations of interest. These PSCs can then undertake a directed differentiation to retinal tissues in vitro, specifically retinal organoids and retinal pigment epithelium (RPE), giving the investigator an abundance of disease‐relevant tissue to assay. There are several applications for the use of retinal organoids and RPE in the scientific study including disease modelling, understanding retinogenesis, drug screening and toxicological screening. This review aims to serve as a primer in the utility of retinal organoids and RPE, derived from PSCs, in the study of inherited retinopathies. Using retinal diseases, such as Retinitis pigmentosa, Stargardt disease and Retinoblastoma, we discuss how PSC‐derived retinal tissues can corroborate results from animal models and uncover novel molecular signatures of disease that may be crucial in developing therapies against inherited blindness.

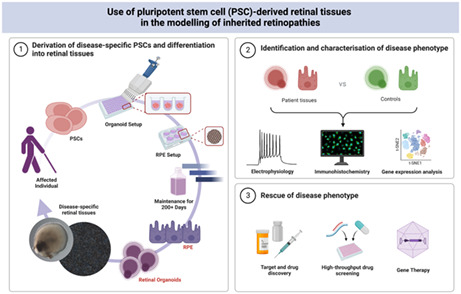

## 1 INTRODUCTION

Existing retinal disease models are largely inadequate for the study of inherited retinal disease (IRD) during retinogenesis in utero. Animal models, particularly murine, have proven useful in informing us of conserved developmental processes across species. However, even though a large proportion of functional DNA sequences are highly conserved between mice and humans, the regulation and expression of these genes differ significantly, limiting their application in the study of human health and disease (Yue et al., 2014). Initiatives such as the Human Developmental Biology Resource (HDBR) have been set up in the UK and aim to address this gap in knowledge by utilising human embryonic and foetal tissue to better understand the pathology underlying the development of congenital anomalies. However, these high‐demand specimens are limited in their use due to a lack of scale for multiple experiments and unsuitability in functional studies (Gerrelli et al., 2015). In the context of IRDs, this issue is further confounded by a high degree of genetic and phenotypic heterogeneity. Clinical presentation often occurs postnatally between early childhood to adulthood (Ellingford et al., 2020). Even with in utero genetic screening, particularly in families with a history of IRDs, these disorders are rarely lethal making it virtually impossible to obtain relevant foetal and embryonic samples through HDBR (Méjécase et al., 2020).

The generation of induced pluripotent stem cells (iPSCs) from human somatic cells has greatly expanded opportunities for disease modelling, particularly in a patient‐specific context. These iPSCs are almost undisguisable from embryonic stem cells (ESCs) and retain the capability to become any cell type specified (Takahashi et al., 2007). With these pluripotent stem cells (PSCs), many research groups have worked to develop in vitro models of retinal cells to better understand human retinogenesis and to model retinal disease (Capowski et al., 2019; Cowan et al., 2020; Dorgau et al., 2019; Hallam et al., 2018; Kuwahara et al., 2019; Mellough et al., 2015; Nakano et al., 2012; West et al., 2022; Zerti et al., 2020; Zhong et al., 2014).

These models can recapitulate in vivo retinogenesis and restate many disease phenotypes seen in patients. They are validated for use in disease modelling by their ability to corroborate findings in animal models, in addition to shedding light on novel pathomechanisms of disease. They have also proven to be useful in the identification of novel therapeutic targets to ameliorate and prevent vision loss. In this review, we discuss several novel insights gained from iPSC‐derived retinal models in the context of IRD pathogenesis. We draw specifically upon the conclusions made using models of three different IRDs that are of particular interest to our own research group. These include PRPF31‐mediated Retinitis Pigmentosa (RP11), Stargardt disease (STGD1) and Retinoblastoma (Rb). Evidence from the studies discussed in this review highlight the importance of iPSC‐derived retinal organoids and RPE in the apprehension of molecular signatures of disease during retinal development in utero which may inform future health screening, diagnoses and prognoses as well as potential prevention measures for vision loss.

## 2 THE MODEL—RETINAL ORGANOIDS

With the use of PSCs, retinal organoids (ROs) or ‘mini‐retinas’ can be generated in vitro and remain stable for over 210 days in suspension culture. These 3D structures are highly reminiscent of the native retina which up to 38 weeks, develop at similar rates in utero (Cowan et al., 2020). ROs mimic the human retinal architecture with apical‐basal polarity as seen in vivo with the development of key neuronal subtypes – rod and cone photoreceptors, bipolar cells, retinal ganglion cells (RGCs), amacrine cells, horizontal cells and Muller glia cells—ordered in a laminated structure. This model offers a unique insight into real‐time retinogenesis, which is more biologically relevant to human physiology than existing in vivo animal models (Collin et al., 2019).

In vivo retinogenesis begins with the establishment of the eye field from the medial anterior neural plate. This is facilitated by the expression of key transcription factors: *Pax6*, *Rax*, *Six3*, *Six6* and *Lhx2* that specify the eye field fate. These transcription factors are also involved in forebrain development. A critical stage in this process is the separation of the eye field from the diencephalon, the caudal region of the forebrain. This is achieved through the evagination of the diencephalon to form optic vesicles (OVs). OVs continue to grow towards the surface ectoderm, which upon contact forms the lens placode. The resulting optic stalk attaching the OVs to the forebrain later becomes the optic nerve (Quinn & Wijnholds, 2019). Invagination of the lens placode and OVs split the eye field in two and generate double‐layered bilateral optic cups. The lens placode continues to develop into the lens vesicle at the surface ectoderm, while the iris and ciliary body begin to form at the anterior of the optic cup. At the posterior, the outer layer of the optic cup becomes a monolayer of hexagonal pigmented cells denoted the retinal pigment epithelium (RPE). Within the inner layer of the posterior optic cup, retinal progenitor cells (RPCs) proliferate to form the intricate network of sensory neurons responsible for the process of phototransduction. The laminated order of the cells present in the retina corresponds to their sequential birth order. RGCs are the first to be generated and the Muller glial cells are last. Asymmetrical division from RPCs produces both post‐mitotic neurons that migrate to their site of function, and additional RPCs continue to populate the retina with a diverse set of retinal neurons (Heavner & Pevny, 2012). ROs mimic the stages of retinogenesis that are specific for neural retina formation. They do not represent the complete ocular system and are therefore not fully representative of the human eye (Nakano et al., 2012). In vivo, the retina forms alongside the lens and accessory tissues. However, this is not the case at present for in vitro retinogenesis.

Current protocols allow for the retina to form as floating spheroids that maintain the structural ordering of key retinal neurons and nascent development of photoreceptor outer segments (POS). However, this culturing method does not support the formation of RPE, a pigmented monolayer adjacent to the photoreceptors. RPE is crucial for the maturation of retinal neurons as well as for phototransduction with the recycling of proteins necessary for the visual cycle (Strauss, 2005). In diseases, where both neural retina and RPE are affected, iPSC‐derived ROs are usually complemented by iPSC‐derived RPE in parallel to obtain insights into the effect of disease on both tissues, often in a patient‐specific context.

**FIGURE 1:**
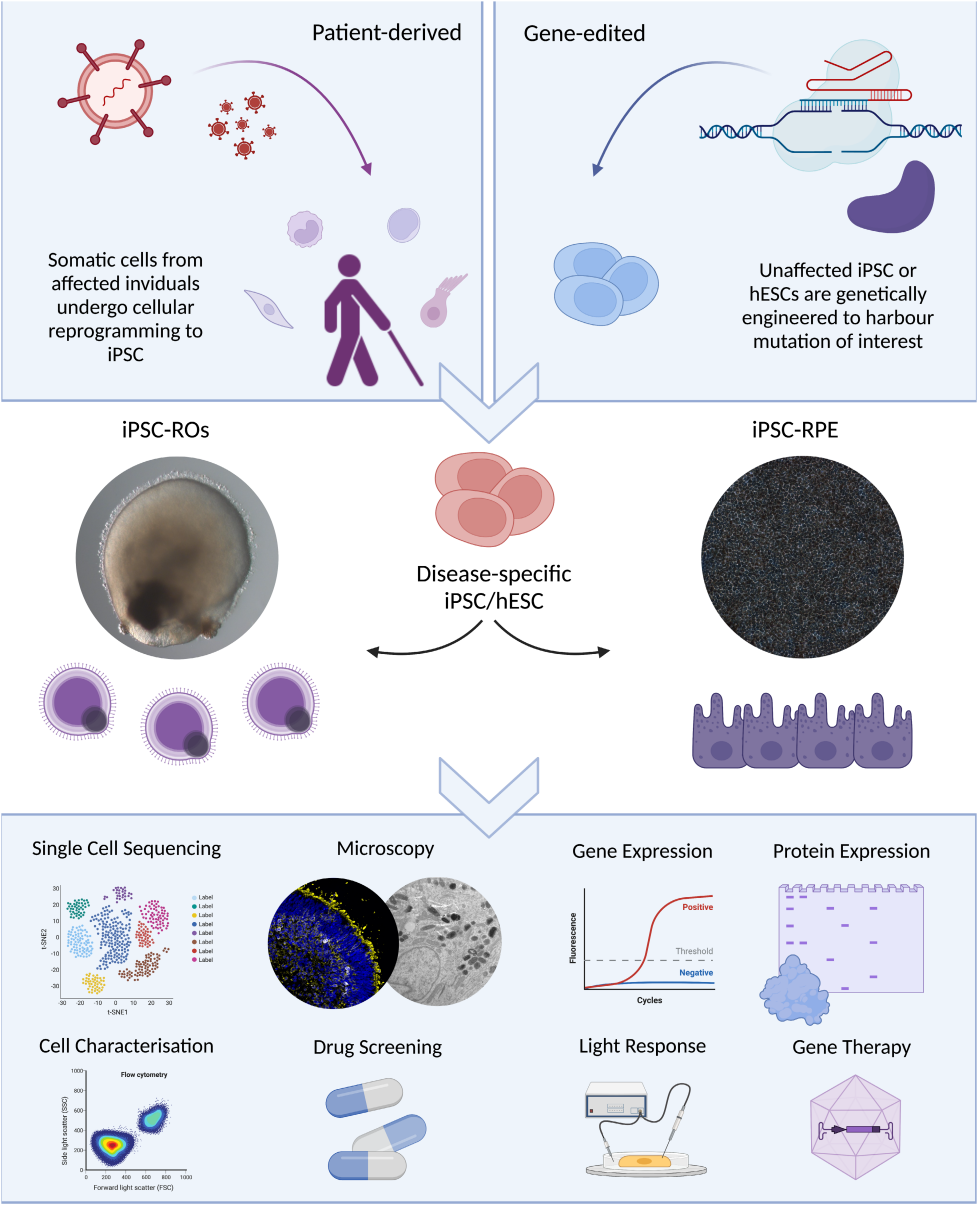
Generation and application of iPSC‐RO and iPSC‐RPE. PSCs can be either induced from somatic cells of affected individuals to generate iPSCs, or genetically engineered using gene editing approaches to introduce relevant mutations into existing PSC lines. The resulting stem cells can undergo differentiation to retinal organoid or RPE tissues for the study of disease in a patient‐specific context. These PSC‐derived tissues can be assayed via a myriad of laboratory techniques to understand the pathogenesis of retinal disease throughout retinogenesis on a molecular level.

Despite evident anatomical inadequacies, past studies have shown the true utility of the RO model in understanding the overarching developmental pathways involved in generating retinal tissue (Mao et al., 2019) as well as the cell‐specific effects of IRD. When taking the latter into consideration, ROs also provide a platform to assess the efficacy and toxicological tolerance of novel therapeutics in early stage proof of concept studies. Their potential within the drug screening and toxicology sector is now also being realised commercially. This is largely due to their scalability in 96‐well plate or pooling formats (Hallam et al., 2018), in addition to their ease of access culturing system, which for other organoid models has already been automated (Renner et al., 2020; Schuster et al., 2020). In vitro retinal models are becoming more useful in the drug‐screening process for biopharma, particularly in the early stages of development. It is known that up to 54% of putative drugs are dropped from phase 3 clinical trials due to an evident lack of efficacy (Hwang et al., 2016). Often this relates back to the initial screening of compounds being tested in ill‐suited, physiologically irrelevant models (Nolan, 2007). According to the FDA, up to 90% of drugs tested on animals fail, and up to 92% of drugs fail in clinical trials after successfully passing pre‐clinical animal studies (Akhtar, 2015). Retinal organoids offer unique insights into the effects of disease‐causing mutations in retinal disease within the developing retina for both the early and late stages of retinogenesis. This is rarely feasible with in vivo animal models and post‐mortem foetal tissue, especially in patient‐specific circumstances. This highlights the importance of ROs as a tool for studying different inherited retinopathies, which to date have very limited therapeutic options available (see Figure 1).

## 3 INHERITED RETINOPATHIES

A significant proportion of retinopathies are attributed to genetic factors and are heritable. These so‐called inherited retinal degenerative disorders are a group of genetically and phenotypically diverse blinding disorders. They affect approximately 1:2000 individuals globally (Berger et al., 2010), making them the most prominent cause of blindness amongst working‐age individuals. Individual IRDs are rare—retinitis pigmentosa (RP), the most common IRD, has an overall incidence of 1:4000 (Pagon, 1988), followed by Stargardt disease (STGD1) with an incidence of 1:8000–10,000 (Blacharski, 1988). The rarity of such conditions was highlighted in a 2019 study that reported more than 20,000 individuals are currently living with inherited vision loss in the UK. This corresponds roughly to 0.0003% of the population that year. Despite their seemingly low frequency, there is a high socio‐economic burden associated with these conditions, which accumulated to £523.3 million that year (Galvin et al., 2020).

**FIGURE 2:**
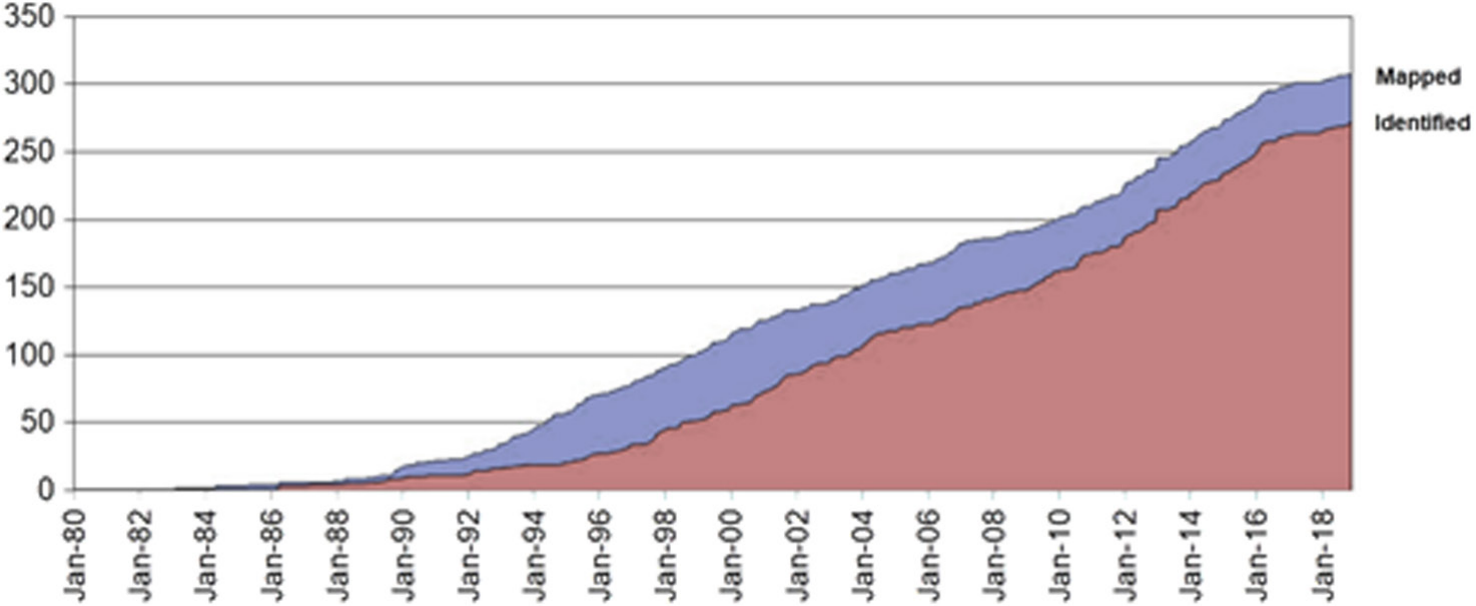
Mapped and identified retinal disease genes. Improvements to molecular diagnostics and research into inherited retinopathies has revealed a high degree of genetic variability in these conditions. More 300 genes have been implicated in the development of inherited retinopathies to date. Figure taken from RetNet (https://sph.uth.edu/retnet/sum‐dis.htm)

To date, approximately 20 individual inherited retinopathies exist with more than 300 genes implicated in their development—see Figure 2 (RetNet, 1998). In the UK, the most frequently mutated genes associated with IRDs in order of highest to lowest incidence are as follows: *ABCA4*, *USH2A*, *RPGR*, *PRPH2*, *BEST1*, *RS1*, *RP1*, *RHO*, *CHM*, *CRB1*, *PRPF31*, *MYO7A*, *OPA1*, *CNGB3*, *RPE65*, *EYS*, *GUCY2D*, *PROM1*, *CNGA3* and *RDH12* (Pontikos et al., 2020). It is apparent that mutations in an array of different retinal disease genes can yield similar clinical phenotypes. This can make definitive clinical diagnoses challenging to attain. Despite molecular differences in the causative underlying pathomechanisms of disease, the majority of IRDs culminate with the degeneration of photoreceptor cells. This results in visual deficits that develop from a relatively young age (early childhood/early adulthood). As photoreceptor cells are postmitotic neurons, there are very few treatment options available once these cells degenerate. Recently, optogenetic reprogramming of RGCsvia AAV delivery of engineered chromophores combined with the provision of computerised visual aids have shown promise in restoring partial vision response in an individual with RP but therapeutic approaches such as this are very much in infancy (Sahel et al., 2021).

Therapeutic strategies should be focused on early intervention rather than towards the end stage of the disease as it is more likely that vision loss can be impeded rather than restored once lost. Ideally, therapies should also target the underlying molecular defect for each retinal disease. However, the heterogeneity combined with the rarity of these IRDs significantly hinders the development of viable therapeutic options for each case. Despite these challenges, the first gene therapy to restore vision in individuals with Leber's congenital amaurosis (LCA) was granted FDA approval in recent years (FDA, 2017). This is a major success in the retinal research field. Vortigene neparvovec‐rzyl, commercially known as Luxturna, restores vision loss caused by biallelic mutations in *RPE65*. RPE65 is important for the recycling of retinal chromophores in the visual cycle. Thereby, defects in this protein compromise PR function, leading to their degeneration and subsequent loss of vision. Luxturna works to deliver the instructions to develop functional copies of this defective protein directly to the retina via AAV vectors allowing for restoration in the recycling of visual proteins and subsequent resumption of visual function (Patel et al., 2016). There are still many gaps in our knowledge that need to be addressed to make therapies more widely accessible and easily adaptable to individual cases of the disease, regardless of their unique genetic background. Mutation‐independent therapeutic strategies to slow down degeneration would be a promising strategy that would be widely applicable to several IRDs. However, little‐known successes have been reported in these approaches to date.

iPSC‐derived retinal models have the potential to offer us patient‐specific insights into the pathophysiology of every retinal disease in a cell‐specific manner. Using these tools, we can track the development of individual cells from the moment they are born until 38 weeks of comparable in utero retinogenesis. This enables us to dissect the influence of genetic mutations in retinal genes on functional networks in the retina to comprehend pathomechanisms of disease and uncover novel therapeutic targets. This literature review discusses three examples of retinal disease, where in vitro iPSC‐derived retinal models have been effective in broadening our knowledge of the retinal disease.

## 4 RP FROM PRPF31 MUTATIONS

### 4.1 Aetiology of the disease

Retinitis Pigmentosa (OMIM #268000) is one of the most prevalent IRDs with an incidence of 1:4000 individuals worldwide (Hartong et al., 2006). In 20%–30% of cases, a secondary disorder accompanies vision loss and is referred to as syndromic RP. Usher syndrome is the most common form of syndromic RP and is characterised by deafness alongside visual deficiencies. RP is a highly heterogeneous disorder implicating more than 70 genes in its pathogenesis with several modes of inheritance including autosomal dominant (adRP) and recessive (arRP), X‐linked and maternal inheritance through mitochondrial DNA (RetNet, 1998). RP displays a wide time frame of disease onset spanning from infancy to adulthood. The disease typically manifests with night blindness and constriction of the peripheral field of vision commonly described as ‘tunnel vision’ which worsens over time and frequently advances to complete blindness in later stages. This is due to the progressive loss of rod photoreceptors and/or RPE (Hamel, 2006).

### 4.2 About PRPFs

Autosomal‐dominant RP is linked to 31 different disease genes and represents 20%–25% of overall RP cases (Hamel, 2006; RetNet, 1998). *P*re‐m*R*NA *P*rocessing *F*actors (PRPFs) are amongst those genes implicated in adRP representing 15%–20% of cases in this cohort of RP. PRPFs are small nuclear ribonucleoproteins (snRNPs) that have a role in the formation and maturation of the spliceosome, a large macromolecular complex involved in the removal of introns and processing of pre‐mRNA to mature mRNA. PRPFs that have been associated with adRP, namely PRPF3, PRPF4, PRPF6, PRPF8, PRPF31, SNRNP200 and RP9, are involved in the formation of the tri‐snRNP, an essential element for the formation of the pre‐catalytic subunit (complex B) of the spliceosome. Mutations in the components of tri‐snRNP reduce the efficiency of spliceosome assembly and result in global dysregulation of splicing (Růžičková & Staněk, 2017).

Despite the ubiquitous expression of PRPFs, it remains enigmatic why the disease phenotype is restricted to retinal tissues. One potential explanation relates to the retina being one of the highest metabolically active tissues in the body. Such high activity poses a significant burden on local splicing machinery that is disparate from any other tissue in the body. Thereby, if the splicing machinery becomes defective, the retina is at a much higher risk of mis‐splicing events causing differential gene expression and subsequent protein dysfunction (Yang et al., 2021).

However, it is difficult to investigate these theories as there is a lack of suitable PRPF disease models. Heterozygote murine models fail to recapitulate the human disease phenotype, although RPE defects are apparent (Bujakowska et al., 2009; Farkas et al., 2014; Graziotto et al., 2011). This is interesting as it suggests that RPE cells are the primary affected cell type in mice. However, given the lack of retinal degeneration in these mice, they are not ideal PRPF‐disease models. This highlights the need to develop disease‐specific PRPF models that can recapitulate human disease, as is possible with the use of human iPSC‐derived tissues.

#### 4.2.1 RP11—PRPF31

##### Morphological and functional defects

**FIGURE 3:**
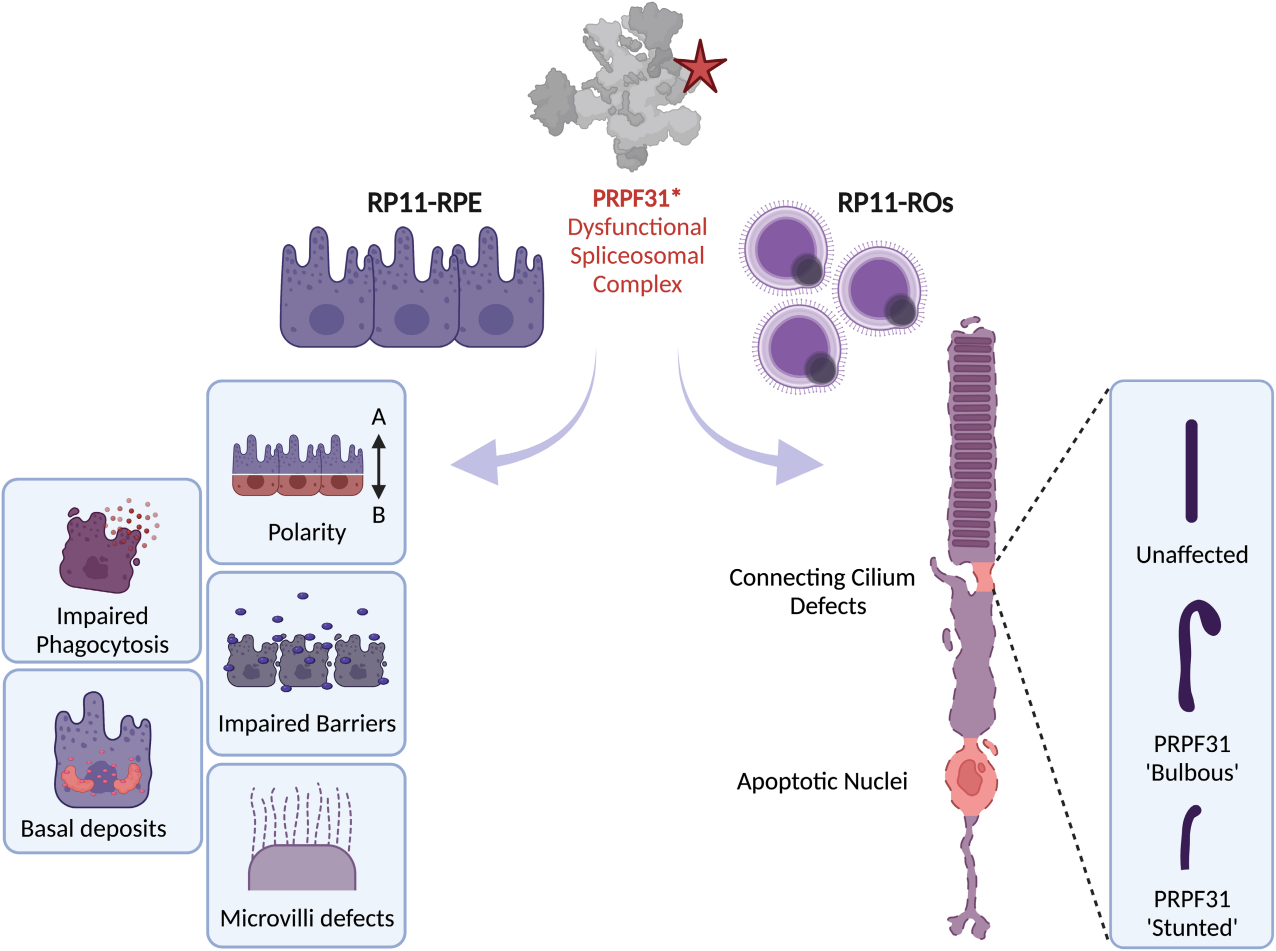
Summary of disease phenotypes observed in stem cell‐derived RP11‐retinal tissues. *PRPF31* mutations negatively impact spliceosome assembly leading to global splicing dysregulation. RP11‐RPE and RP11‐ROs were derived from patient iPSCs and revealed defects in molecular structures. RP11‐RPE reveals several defects including disrupted apical/basal polarity and tight junctions, altered microvilli morphology, defective phagocytotic function and the appearance of basal deposits. RP11‐ROs harboured photoreceptor cells with apoptotic nuclei and altered connecting cilium. Figure adapted from Buskin et al. (2018).

In recent years, our research has been focused on exploring the molecular pathogenesis of adRP caused by mutations in *PRPF31* (RP11), a gene which encodes the PRPF31 protein, which is involved in U4/U6 snRNPs assembly (Makarova et al., 2002). Using PSC technology, iPSCs from patients carrying severe (c.522_527+10del) and very severe (c.1115_1125del11) mutations were generated and differentiated into retinal organoids (RP11‐ROs) and RPE (RP11‐RPE; Buskin et al., 2018). RP11‐RPE displayed the most significant disease phenotype indicating that RPE may be the primary cell type affected by PRPF31‐mediated disease. A result that corroborates findings in heterozygote murine models (Farkas et al., 2014). The disease phenotype of these cells was characterised by the loss of apical‐basal polarity as described by impaired tight epithelial barriers, deformed microvilli which were both shorter and fewer in incidence, defective phagocytic function, and the occurrence of large basal deposits. Defects in phagocytic function have been reported previously in mouse models of PRPF31^+/−^ accompanied by abnormal retinal adhesion but did not show any defects to the neural retina (Farkas et al., 2014). However, RP11‐RO‐derived photoreceptors did display altered morphology with a 150% increase of apoptotic nuclei compared with controls. This was accompanied by the presence of stress vacuoles which is a characteristic feature of adaptive survival. Such a defect has not been reported previously. RP11‐ROs also displayed a reduced RGC spiking rate in response to the neurotransmitter GABA on multi‐electrode arrays (MEAs). Measuring RGC activity is used as an indication of neural connectivity between retinal cells in the organoids. Reduced activity suggests impaired connectivity of emerging retinal networks in the RP11‐ROs—see Figure 3 (Buskin et al., 2018).

##### Splicing defects

Splicing defects were present in RP11‐ROs and RP11‐RPE yet absent from iPSCs and fibroblasts carrying the same mutation, confirming the restriction of PRPF31‐mediated mis‐splicing to retinal tissue. The most affected genes in these tissues determined via gene ontology (GO) were related to pre‐mRNA and alternative mRNA splicing. Interestingly, the identification of long‐mutant *PRPF31* isoform was present in RP11‐RPE but not in RP11‐ROs. In addition to this, RP11‐RPE also displayed the most significant differential exon usage with many transcripts retaining introns and using alternative 3′ splice sites, further suggesting that RPE cells are the most affected cell type (Buskin et al., 2018).

In the same study, GO enrichment for RP11‐fibroblasts also showed significant differential exon usage for genes involved in ciliogenesis and maintenance, validating previous work which showed reduced cilia length and incidence in fibroblasts from RP11 patients (Wheway et al., 2015). A similar phenotype was seen in RP11‐ROs and RP11‐RPE with cilia described as defective and bulbous with misaligned microtubules. The association of this phenotype with *PRPF31* mutation was confirmed with siRNA knockdown in a human RPE cell line which recapitulated the disease phenotype.

With the use of CRISPR/Cas9 technology, the most severe RP11 variant in this study (RP11VS) was corrected and then differentiated alongside RP11‐iPSCs. Genetic correction of this defect restored the phenotype of retinal cells with increased cilia length and incidence in both photoreceptors and RPE cells. Phagocytotic capacity of RPE cells was also restored along with apical‐basal polarity. This demonstrates that CRISPR/Cas9 gene editing in situ is a possible route to explore for therapeutic intervention of PRPF‐mediated retinal disease (Buskin et al., 2018). In addition to this, gene augmentation strategies using AAV vectors to deliver functional copies of *PRPF31* to RP11‐RPE derived from the same RP11‐iPSCs also significantly ameliorated the phenotype observed in diseased cells (Brydon et al., 2019).

This study was the first of its kind to model RP11 with iPSC‐derived retinal cells and indeed proved indispensable for gaining novel molecular and developmental insights into PRPF31‐mediated pathogenesis. With the use of this model, several previous findings have been corroborated, such as the observation of RPE being the primary cell affected in this disease (Farkas et al., 2014). The reduced expression of wild‐type PRPF31 in both iPSC‐derived RP11‐RPE cells and RP11‐photoreceptors confirms previous hypotheses of haploinsufficiency as the mechanism of pathogenesis in RP11 patients (Dong et al., 2013; Rio Frio et al., 2008; Rivolta et al., 2006).

These results validate the use of iPSC‐derived retinal tissues as a tool to investigate and understand the full pathological mechanisms underlying RP11. In RP11‐RPE, a number of molecular defects were observed and represent further lines of research constituting potential therapeutic targets in the future. Indeed, a recently published continuation of this study from our group has provided a more in‐depth analysis of these defects and has demonstrated that the long‐mutant isoform of *PRPF31* does not act in a dominant‐negative manner to elicit retinal degeneration in RP11 (Georgiou et al., 2022). This finding has previously been observed in vivo with the *Prpf31* ^*A216P/+*^ mouse model (Bujakowska et al., 2009) and further demonstrates the utility of iPSC‐derived retinal models in the study of RP11 and other inherited retinopathies.

##### Basal deposits

The appearance of basal deposits on RP11‐RPE is an interesting phenotype as misfolded proteins tend to aggregate and deposit and are characteristics of many degenerative disorders. In addition to this, other PRPFs, namely PRPF3, are known to form aggregates with increased propensity to form insoluble proteins (Comitato et al., 2007). However, the underlying mechanism is poorly understood. In a recent paper, the *Prpf31* ^*A216P/+*^ mouse model displayed similar aggregation which localised to the cytoplasm of RPE cells. This was accompanied by an upregulation of heat shock protein A4L (*Hspa4l*), a molecular chaperone involved in the repair of misfolded proteins (Valdés‐Sánchez et al., 2019).

**FIGURE 4:**
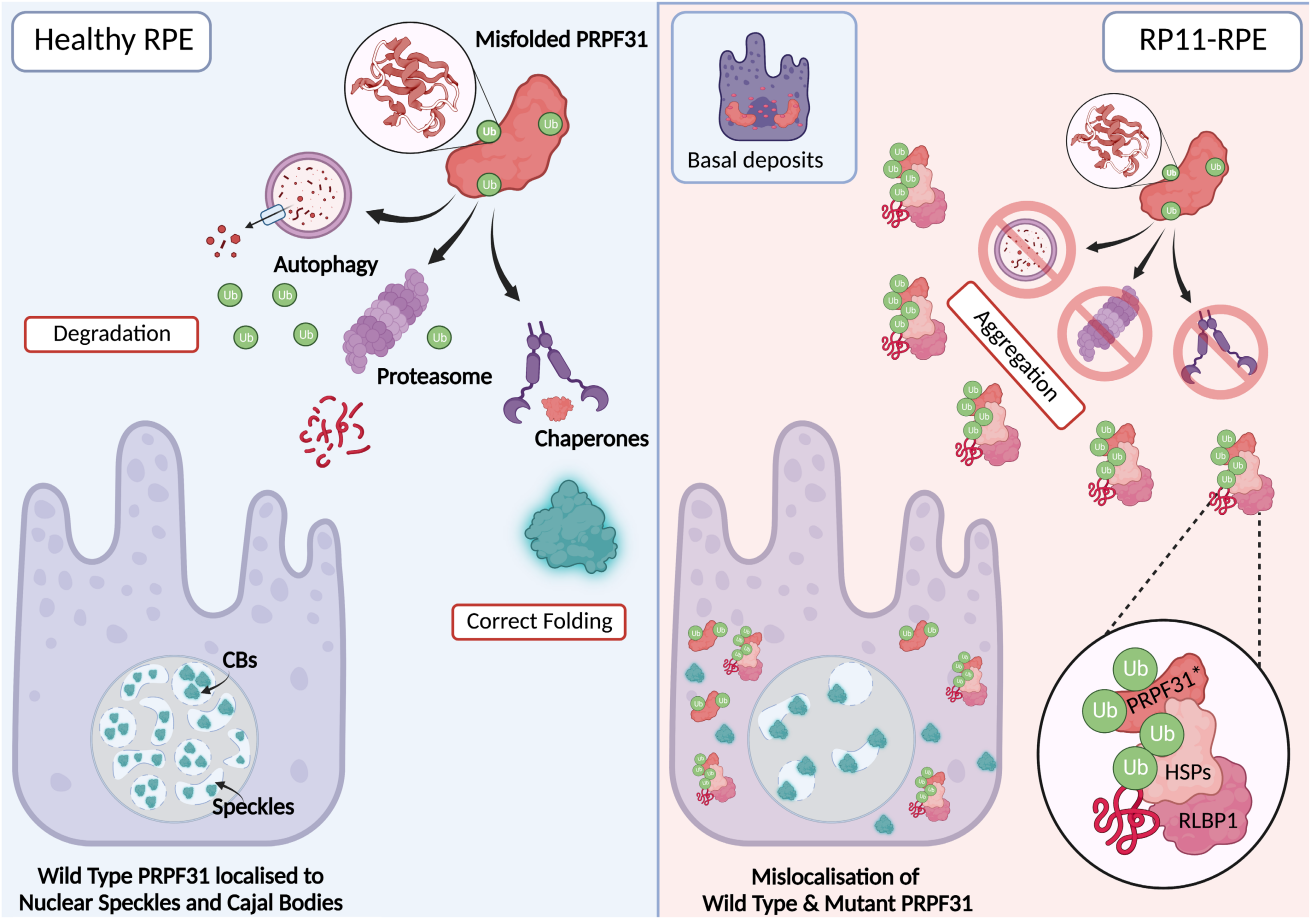
Mechanism of aggregate formation in RP11‐RPE. There are several responses to misfolded proteins in healthy RPE. Molecular chaperones such as heat shock proteins (HSPs) can be employed to stabilise the protein and assist it in reaching its correct conformation. If the protein is too severely misfolded, the protein is tagged with ubiquitin molecules targeting it for degradation. This can occur either via the proteasome or via a process known as autophagy. These processes recycle the components of the misfolded protein. In the case of misfolded PRPF31, these processes ensure that no defective protein is produced and is correctly localised to the Cajal bodies (CBs) and nuclear speckles within the nucleus of the cell. In RP11‐RPE, unfolded protein response (UPR) pathways are impaired, likely due to mis‐splicing events because of defective PRPF31 protein. This results in the formation of cytoplasmic aggregates comprised of mutant PRPF31, mutant HSPs, and additional mutant retinal proteins such as RLBP1 which negatively impact the health of the cell. These defects also appear to direct the localisation of both mutant and wild type PRPF31 protein to the cytoplasm of RPE cells. Figure adapted from Georgiou et al. (2022).

Using the RP11‐RPE and RP11‐ROs as described in the 2018 Buskin et al. paper, a more thorough analysis of the basal deposits was carried out (Georgiou et al., 2022). This revealed the mislocalisation of PRPF31 protein to the cytoplasm of RP11‐RPE cells in association with other misfolded and ubiquitinated proteins tagged for proteasomal degradation, implicating mutant PRPF31 in the aggregation process occurring in RP11‐RPE cells—see Figure 4.

While wild‐type PRPF31 can be found in the insoluble fractions of RP11‐RPE and isogenic controls, long‐mutant isoform *PRPF31* can only be seen in fractions from RP11‐RPE. A reduction in wild‐type PRPF31 was also evident in the soluble fractions of RP11‐RPE suggesting the involvement of mutant PRPF31 in the aggregation occurring in the cytoplasm. The presence of wild‐type PRPF31 present in the cytoplasm can be explained by its association with nuclear speckles. Nuclear speckles are organelles present in the cytoplasm and are known to accumulate snRNPs to mediate post‐transcriptional modification (Spector & Lamond, 2011). In fact, in isogenic control RPE tissues, PRPF31 co‐localised with SC35, a marker of nuclear speckles. However, in RP11‐RPE, a substantial proportion of PRPF31 did not co‐localise with SC35. A similar phenotype was observed in RP11‐RO‐derived photoreceptor cells.

Cajal bodies, a region of the nucleolus, are the site of tri‐snRNP assembly (Carmo‐Fonseca et al., 1991). RNA‐FISH experiments for snRNAs U4, U6 and U5 alongside coilin (a marker of Cajal bodies) were conducted to investigate if mutant PRPF31 was directly influencing the assembly of tri‐snRNP in Cajal bodies of photoreceptors and subsequently if complex B formation is disrupted. An accumulation of U5 snRNA alongside reduced U4 and U6 snRNAs was observed as well as a generalised accumulation of U snRNAs on Northern blots from cell extracts of RP11‐RPE and RP11‐ROs. Importantly, while sedimentation patterns in Western blotting of lysates from RP11‐RPE were abnormal, no association of mutant PRPF31 with these snRNAs was observed as evidenced by the presence of a single band on the blot corresponding to mutant PRPF31. This suggests that mutant PRPF31 does not directly inhibit complex B formation. However, the reduction in wild‐type PRPF31 expression and ensuing mis‐splicing of other splicing factors results in a global spliceosome dysregulation that is specific to RPE and retinal cells (Buskin et al., 2018).

#### 4.2.2 Proteomics

Mutations in *PRPF31* are known to cause a global impact on the splicing of genes involved in the structure and function of retina‐specific cells (Tanackovic et al., 2011). Quantitative proteomics data obtained from our group's study corroborates this and reveals the major pathways affected in RP11‐RPE and RP11‐ROs compared with isogenic control tissues.

#### 4.2.3 RP11‐RPE

In RP11‐RPE, GO enrichment analysis revealed several categories of differentially expressed genes in processes such as RNA splicing, spliceosome complex, retinoid metabolism, visual perception, lysosome and protein folding pathways. FUS protein was the most downregulated protein in the RNA splicing pathway and showed aggregation in the cytoplasm of RP11‐RPE. RLBP1 was the most significantly upregulated protein in the visual perception pathway and was also prone to aggregation, particularly at the tight junctions between cells. The protein folding pathway showed upregulation of two molecular chaperones: HSPB1 and HSPA2, which co‐localised with a proportion of cytoplasmic aggregates. These proteins were also upregulated in RP11‐ROs suggesting a similar mechanism of unfolded protein response (UPR) in the neural retina.

It was clear that the cytoplasmic aggregates identified in RP11‐RPE were destined for degradation via the UPR pathway by the upregulation of FK1—a marker of ubiquitinated proteins, in the patient cell lines. Transmission electron microscopy (TEM) revealed the presence of multivesicular bodies and large vacuoles filled with electrodense material in RP11‐RPE. Evidence of debris was also observed in gaps between the cells suggesting these aggregates contribute to the disruption of tight junctions between cells.

#### 4.2.4 RP11‐ROs

In RP11‐ROs, a significantly high proportion of differentially expressed genes were identified. However, only those related to PRPF31 aggregation and photoreceptor function were considered. These included ER lumen, autophagy and lysosome, retinoid metabolism, visual perception, response to ER‐stress and UPR pathways. Generally, PRPF31 and numerous other snRNPs were significantly downregulated in RP11‐ROs. Particularly, NOVA1 and PTBP2, which are proteins involved in the modulation of neuronal‐specific genes in the brain and are vital for neuronal survival, were amongst the most significantly downregulated. This suggests defective mRNA metabolism in RP11‐ROs (Jensen et al., 2000; Li et al., 2014).

Rab GTPases are described as central coordinators in membrane trafficking in the autophagy processes. They have roles in the regulation of membrane tethering, movement and fate (Jin et al., 2021). Interestingly, in RP11‐ROs, Rab27a and Rab38 showed a threefold increase in expression compared with controls. This is indicative of the activation of autophagy.

In the context of misfolded proteins, the first line of defence is molecular chaperones to assist in the stabilisation of folding intermediates to prevent the formation of defective protein or aggregation of said protein (Hartl et al., 2011). If the production of a stable protein cannot be achieved by the chaperones, the peptide sequence is ubiquitinated and tagged for proteasomal degradation. However, if the proteaosome becomes overwhelmed and malfunctions, autophagy is activated.

Autophagy is an innate process where the cell performs self‐phagocytosis to eliminate misfolded proteins and damaged organelles in response to endoplasmic reticulum (ER) stress. In normal scenarios, this mechanism is tightly transcriptionally regulated. However, if stresses are prolonged and acute, this can result in significant cell death (Deegan et al., 2013). This is an issue particularly for neuronal tissue where most cells are post‐mitotic and not capable of regeneration.

In the lysosomal pathway, LAMP1 and LAMP2 were significantly upregulated. These proteins are involved in the fusion process of phagosome and lysosome. As components of the lysosomal membrane that constitute 50% of total protein in the lysosome, their function is extremely important and their upregulation is often associated with the initiation of autophagy (Eskelinen, 2006). This suggests that autophagy has been activated in these cells because of increased mis‐splicing events and subsequent protein aggregation in RP11‐ROs.

#### 4.2.5 Autophagy

Several proteins involved in the proteasomal degradation pathway including chymotrypsin‐like (PSMB8), caspase‐like (PSMB9) and trypsin‐like (PSMB10) were significantly downregulated suggesting impaired proteasomal degradation in RP11‐RPE. As autophagy is often initiated in these scenarios, the expression of markers in each stage of autophagy was assessed. This revealed a block in late‐stage autophagy with the upregulation of several proteins including p62 and LC3‐II. These are conventionally associated with an inhibition of autophagosome degradation (Tanida et al., 2004).

By performing autophagy flux assays in the presence of Bafilomycin—a drug known to block the autophagy pathway by inhibiting autophagosome‐lysosome fusion in vitro (Xie et al., 2014), it was true that the process of autophagy was inhibited in RP11‐RPE. In the presence of Bafilomycin, the expression of autophagy‐related proteins like LC3‐II and p62 should increase as the catabolic process of autophagy cannot occur and associated proteins are not degraded.

Expectedly, an increase of autophagy‐related proteins was observed in isogenic control tissues in the presence of Bafilomycin, but not in RP11‐RPE suggesting impairment of autophagy. When seen in other studies, this is often associated with the activation of the mTOR pathway (Mauvezin & Neufeld, 2015). Daily feeding of the RP11‐RPE with rod POS accelerated the formation of aggregates and inhibition of autophagy. The response of which increased local caspase‐3‐mediated apoptosis.

It is known that inhibition of the mTOR pathway activates the autophagy pathway in developing and ageing tissues (Schmeisser & Parker, 2019). Rapamycin has been identified as a potent inhibitor of mTOR signalling and subsequent activator of autophagy (Cao et al., 2006; Ravikumar et al., 2002, 2004).

**FIGURE 5:**
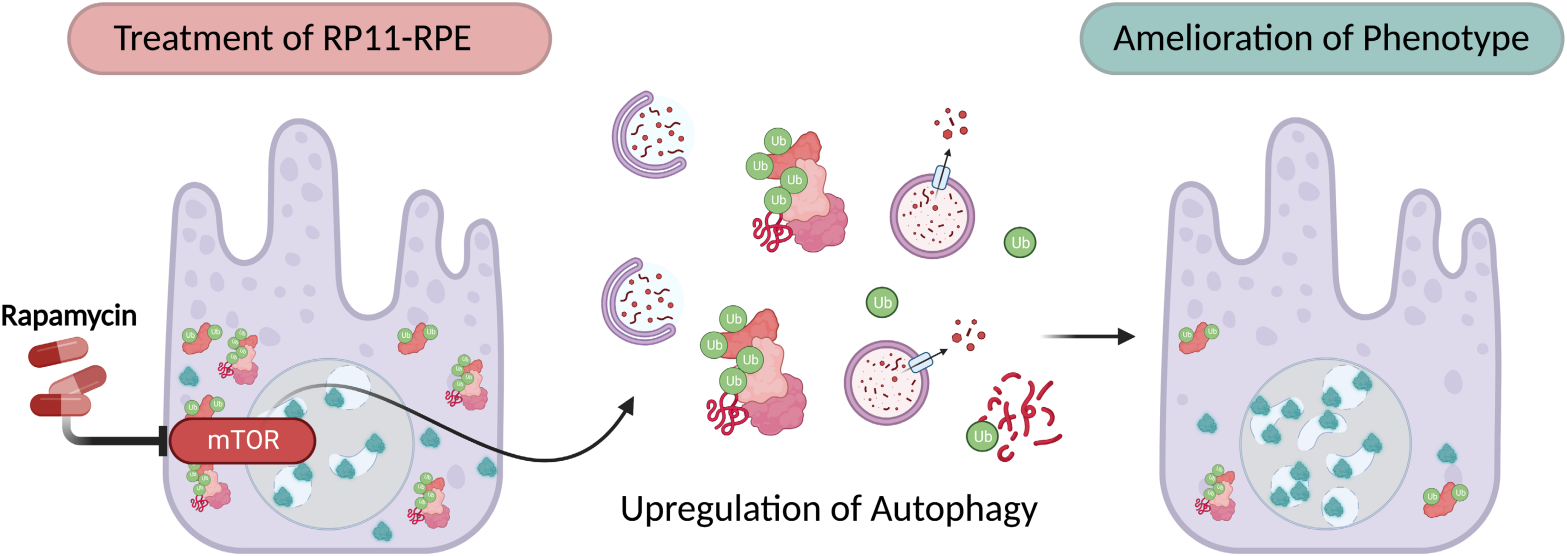
Treatment of RP11‐RPE with rapamycin. Rapamycin inhibits the mTOR pathway, and subsequently upregulates the process of autophagy. Increased activation of autophagy enables degradation of misfolded PRPF31 and associated aggregates. This results in an ameliorated phenotype in RP11‐RPE with reduced cytoplasmic aggregates and mutant PRPF31 in the cytoplasm. Improved localisation of wild type PRPF31 is also observed. Figure adapted from Georgiou et al. (2022).

Culturing RP11‐RPE with Rapamycin for 7 days was sufficient to activate autophagy. This significantly reduced the burden of cytoplasmic aggregates in RP11‐RPE, such as those containing RLBP1 and chaperone protein HSPB1. Reduced activation of caspase‐3 was also observed in the RP11‐RPE and subsequently enhanced cell survival—see Figure 5 (Georgiou et al., 2022). Although an amelioration of disease phenotype was observed via the upregulation of the autophagy pathway, this treatment does not correct the intrinsic issue of defective splicing machinery due to mutated PRPF31 protein and would therefore be insufficient to treat RP11 disease in patients. Realistically, this treatment is targeted towards the deleterious secondary effects of aberrant splicing by assisting in the clearance of cellular aggregates. In the clinic, it would likely be used in combination with a gene augmentation strategy to deliver functional copies of *PRPF31* to correct the defect in the tissue in a manner discussed previously (Brydon et al., 2019).

Overall, these studies highlight the importance of using iPSC‐derived retinal models to elucidate the molecular pathogenesis underlying inherited retinopathies. Phenotypes and processes of pathogenesis identified previously in animal models of RP were successfully recapitulated in RP11‐RPE thereby validating the use of this model for investigating additional molecular signatures of RP11 disease.

These models provided novel insights into the molecular pathogenesis of RP11, with the exact localisation of cellular aggregates in the cytoplasm and the defective processes involved in promoting the accumulation and aggregation of misfolded proteins. This ultimately led to novel targets to exploit for the activation of autophagy and accelerated clearance of cytoplasmic aggregates. The proteomics analyses revealed even further targets such as the downregulated vision‐related proteins that are often associated with other IRDs. This is the first example of an RP11 in vitro model with physiological relevance to humans and has proven to be an invaluable resource for uncovering the pathogenesis of a poorly understood retinal degenerative disorder.

### 4.3 Stargardt disease

Stargardt disease (STGD1; OMIM #248200) is monogenic inherited retinopathy caused by bi‐allelic mutations in the *ABCA4* gene (Allikmets et al., 1997). This gene encodes a large transmembrane protein (255 kDA) and is involved in the clearance and recycling of toxic bisretinoid by‐products of the visual cycle (Molday et al., 2009). Defects in this process lead to inefficient clearance of retinal resulting in the aggregation of bisretinoid molecules, which are later metabolised to lipofuscin. Lipofuscin is described as a post‐mitotic pigment composed of lipid derivatives from the lysosomal degradation pathway. It is a toxic molecule capable of causing cellular stress and subsequently apoptosis (Terman & Brunk, 1998).

STGD1 affects the macular region of the retina and causes a progressive decline in chromatic and central vision as the photoreceptor cells in the central retina and underlying RPE degenerate. There are currently no effective therapies to alleviate or halt symptoms of this disease which tends to present early in adolescence (Tanna et al., 2017). STGD1 is the leading cause of macular blindness in young adults with an incidence of 1 in 8000–10,000 individuals globally (Blacharski, 1988). Realistically, rates are likely much higher than reported due to the high degree of genetic polymorphism within the *ABCA4* gene (Webster et al., 2001).

This condition displays significant clinical and allelic heterogeneity, which impacts the accuracy and ability to correctly diagnose affected individuals based on phenotype alone. With the increased accessibility of next‐generation DNA sequencing, many affected individuals which display the appropriate phenotype can receive an accurate genetic diagnosis of their STGD1 status with up to ~70% success (Khan et al., 2019; Khan, Cornelis, et al., 2020b). However, ~30% of cases are unsuccessful in the identification of conclusive bi‐allelic variants or in some cases, any *ABCA4* variants at all, despite displaying strong clinical characteristics of the disease. The importance of obtaining a genetic diagnosis for these unsolved individuals is highlighted by the requirement for one to enlist in appropriate clinical trials that are ongoing for STGD1 and similar maculopathies. It is also crucial for genetic counselling of the affected individual to inform on their prognosis and risk of transmission to their offspring.

With existing technologies, a substantial proportion of coding variation in *ABCA4* has already been uncovered. This suggests that a potential major source of the missing inheritance in unsolved cases could be due to rare defects in the non‐coding regions of the gene (Zernant et al., 2011). The non‐coding regions of the genome are known to play a crucial role in orchestrating gene splicing and regulating gene expression. Therefore, RNA defects have immense potential to cause aberrant splicing and subsequently have damaging effects on protein structure and function. RNA defects tend to be more difficult than coding variants to detect in DNA sequencing data due to the ineffectiveness of in silico variant pathogenicity algorithms to accurately predict the functional outcome of putative splicing variants. This often results in their exclusion during the variant filtering and prioritization process (Sangermano et al., 2016). Whilst some RNA variants are successfully identified in DNA sequencing, they often must undergo in vitro functional assays to confirm their effect on protein expression (Sangermano et al., 2018).

There have been several studies demonstrating the identification of such RNA defects using augmented screening approaches in previously unsolved *ABCA4‐*mediated retinopathies (Bauwens et al., 2019; Bax et al., 2015; Cornelis et al., 2017; Schulz et al., 2017; Zernant et al., 2018) and in some cases, their validation with functional midigene splicing assays (Albert et al., 2018; Sangermano et al., 2018). Assays for this condition need to occur in tissue‐specific cells due to the unique expression of *ABCA4* in the retina. This can be achieved by gene editing iPSC lines to induce the mutation of interest. The differentiation of these cells to photoreceptor precursor cells (PPCs) allows for insight into the effect of the mutation on the protein. If protein effects are demonstrated by the assay, the unsolved case can be reclassified as solved with high confidence. While PPCs are effective in confirming a splicing defect and serving their purpose in this context, they are not suitable in vitro model to fully characterise the impact of the mutation on other cell types of the retina. PPCs are an immature cell type cultured in isolation in a two‐dimensional format. Along with the loss of physiological context of the retina, ABCA4 is expressed specifically in the photoreceptor outer segment, which takes a minimum of 120–180 days in RO suspension culture to develop (Capowski et al., 2019).

A number of animal models exist for STGD1 with many more in development. The ABCA4^−/−^ murine model of STGD1 has provided several useful insights such as the evident accumulation of the toxic bisretinoid NRPE in the RPE (Weng et al., 1999). Histological sections of post‐mortem ABCA4^−/−^ mouse also reveal the accumulation of lipofuscin that is seen with in vivo imaging of murine retinas (Charbel Issa et al., 2013) supporting ABCA4’s role as a transporter of spent retinal molecules. A critical observation from the ABCA4^−/−^ mouse was the elevation in NRPE accumulation following exposure to blue light and the ingestion of large doses of vitamin A. This is important as dietary supplementation of vitamin A is often associated with reduced risk for age‐related macular degeneration, a condition with a similar phenotypic presentation to STGD1 (de Koning‐Backus et al., 2019). These findings resulted in the recommendation of STGD1‐affected individuals to avoid bright sunlight with sunglasses and avoid foods rich in vitamin A. Despite these crucial discoveries, the mouse does not have the correct anatomy to fully recapitulate the STGD1 phenotype. It is known that the murine retina is largely comprised of rod photoreceptors and lacks a macula, the region with the most affection in STGD1. To this end, several models have been developed to gain more apposite insights into ABCA4‐mediated pathogenesis. These include the pig model (Trapani et al., 2019), zebrafish model (Collery, 2020) and perhaps also a dog model (Le Bras, 2019).

In addition to animal models of disease, iPSC‐derived retinal tissues can also be utilised for the modelling of disease‐causing *ABCA4* mutations. Specific differentiation conditions can be specified to yield retinal organoids with increased populations of cone photoreceptors which may be useful for assessing the effects of ABCA4 deficiencies on the retina in a cell‐specific manner (Zerti et al., 2020). This could be useful as cones appear to be more susceptible to stress in the absence of functional ABCA4 protein in ABCA4^−/−^ NRL^−/−^ mouse models, despite both rods and cones expressing ABCA4 protein (Conley et al., 2012).

A number of groups have already successfully generated iPSC‐derived retinal tissues such as retinal organoids (Su et al., 2021) and RPE (Hu et al., 2020) for STGD1. One study has also utilised STGD1‐ROs in a proof of principle therapeutic experiment to restore an aberrant splicing defect in *ABCA4* (Khan, Arno, et al., 2020a). Aside from that, our own research group is working to model a small subset of STGD1 with late‐onset presentation of clinical phenotypes. These individuals acquire visual defects mid‐life, often due to the phenomenon of foveal sparing. This preservation of foveal tissue is associated with less severe *ABCA4* defects and certainly appears to be mutation specific. Often individuals with late‐onset STGD1 display missing heritability (Westeneng‐van Haaften et al., 2012). Evidence shows that the development of late‐onset STGD1 is likely influenced by modifier effects in regions outside of the functional zones of the ABCA4 protein (Yatsenko et al., 2001). More recently, late‐onset STGD1 has been associated with previously uncovered mild deep intronic variants (Runhart et al., 2019). These novel findings suggest a potential source for the missing heritability in these cases that remain to be solved. Our study focuses on the disease modelling of monoallelic late‐onset STGD1 to produce mature retinal‐specific *ABCA4* RNA transcripts to assess whether mild splicing defects could be causative of disease. This will be achieved through long‐read sequencing so that differential isoforms of *ABCA4* can be compared between patient and wild‐type control tissues.

Although this study is ongoing, to date, we have demonstrated that patient‐derived late‐onset STGD1 ROs develop laminated retinal tissue with the presence of all key retinal neurons. This is supported by gene expression data of retinal markers at various time points throughout differentiation (unpublished results). We matured these organoids until day 230 and detected ABCA4 protein expression in nascent photoreceptor outer segments. We have also confirmed the expression of ABCA4 by Western blotting. With the use of a confirmed biallelic STGD1 control line, we showed reduced protein expression as expected from gene expression data in a study utilising the same iPSC line (Albert et al., 2018).

From our early research, retinal organoids appear to be a suitable and accessible tool to investigate the pathogenesis of both *ABCA4* coding and non‐coding defects as well as the effects photoreceptor degeneration may have on other cell types in the retina. However, as mentioned previously, retinal organoids do have some limitations, and these are particularly important in the context Stargardt disease. The absence of adjacent RPE cells poses the question of whether the underlying classical pathogenesis of STGD1, where lipofuscin accumulates in RPE and secondarily degenerates overlying photoreceptor cells, can occur in the model (Lenis et al., 2018). In principle, defective ABCA4 could still accumulate in the POS and increase stress and apoptosis in photoreceptors. However, this does present difficulties in recapitulating the full clinical phenotype in vitro and cannot be addressed until reliably connected models of RO and RPE are developed and can be co‐cultured. These issues are further compounded by the fact that retinal organoids do not fully mimic the distribution of photoreceptor cells in the retina, meaning that no fovea exists in this model. We have considered the latter in the context of our own studies and our solution, although imperfect, is to increase the number of cone photoreceptors in our model to give a more ‘foveal’ appearance through a protocol devised in our own group (Zerti et al., 2020). Despite this, ROs are the most advanced retinal models that exist in vitro and do hold the highest resemblance to developing human retinas when compared with animal models, so their utility outweighs their limitations.

Having accurate models of STGD1, stratified in mutation and disease severity, is crucial for the holistic understanding of STGD1 and other *ABCA4*‐mediated retinal diseases. In the context of our own work, retinal organoids appear particularly useful for providing RNA transcripts that are physiologically accurate to that of in vivo retina due to the development of iPSC‐derived photoreceptors within a retinal niche. The corresponding data are likely to be the most accurate representation of the true in vivo scenario and could supplement existing data to improve and further train existing splicing algorithms so that the process of classifying RNA defects is more robust as well as serving as a platform for testing ABCA4 functionality, identifying novel therapeutic candidates and for the drug screening process in pre‐clinical studies.

### 4.4 Retinoblastoma

#### 4.4.1 Aetiology

Retinoblastoma (Rb) is an intra‐ocular childhood cancer that initiates in the retina of developing eyes. The overall incidence of this condition is 1:15,000–20,000 globally, accounting for 9000 new cases each year in individuals <5 years of age (Kivelä, 2009). Rb is initially identified when a patient presents with leukocoria—a whiteness in the pupils of the eye. This whiteness is an intraocular tumour occluding view of the retina at the back of the eye. In the early stages, 3–6 months after the development of leukocoria, 5‐year survival rates are high at 96% with the enucleation of affected eyes (Siegel et al., 2022). However, delayed diagnosis and treatment heighten the risk of invasion of this intraocular cancer into the optic nerve and brain, leading to global metastases and subsequently reduced survival rates (Dimaras et al., 2012). Additional symptoms of Rb are misaligned eyes (strabismus), glaucoma, motion processing and local generalised inflammation of the eye.

The genetic basis of cancer was first discussed in the context of Rb which is caused by the biallelic inactivation of tumour suppressor *RB1*. Knudson in 1971 made the observation that Rb formation is dependent on two mutational events taking place in this gene and termed this the ‘two‐hit hypothesis’ (Knudson, 1971).

Rb occurs by both heritable and non‐heritable means. In the heritable form, affected individuals carry a constitutional germline mutation in *Rb1* (Mutation 1) pre‐disposing them to Rb. The second mutation occurs somatically in a susceptible cell of the retina (Mutation 2). In addition to increased risk for Rb development, individuals with constitutional mutation of *Rb1* also have a predisposition to secondary cancers in other tissues (Eng et al., 1993; Roarty et al., 1988). Heritable Rb can present as unilateral (60% cases) and bilateral (40% of cases) disease, whereas non‐heritable forms always present unilaterally. This is due to both Mutation 1 and Mutation 2 occurring somatically in the same susceptible retinal cell (Thériault et al., 2014). In the last decade, a small subset of Rb cases was found to have wild‐type and functional retinoblastoma protein (pRB) and have been linked with increased copy numbers of *MYCN*, a known oncogene. These MYCN cases correspond to 2% of overall Rb cases (Rushlow et al., 2013).

### 4.5 Pathological mechanism

pRB is an important cell cycle regulator that works in concert with cyclin‐dependant kinases (CDKs) to regulate the G1 to S‐phase transition in the cell cycle. Specifically, pRB acts to repress E2F transcription factors which activate genes related to cellular proliferation. In response to mitotic signals in the environment, CDKs hyperphosphorylate pRB to relieve its repression on E2Fs and subsequently permit cell cycle entry. Considering the important role pRB plays in this process, it makes sense that loss of its function leads to uncontrolled cellular proliferation and the initial formation of benign tumours called retinomas. Increased cellular proliferation leads to increased genomic instability and enables the transformation of susceptible cells via the acquisition of secondary mutations in *Rb1*. If left unchecked, retinoblastomas continue to acquire genetic aberrations and become independent. Parts of the tumour break away and invade neighbouring tissues causing metastasis (Dimaras et al., 2015).

Despite the years of research and a number of studies on Rb, it remains an enigma as to why a ubiquitously expressed tumour‐suppressor gene results in a retina‐specific phenotype when defective. It has been suggested that this is likely due to specific characteristics of a susceptible cell in the retina. Identifying this cell has been somewhat challenging but numerous studies have pinpointed the cone precursor as the cell of origin. Early studies consistently saw the expression of key cone cell markers in low passage Rb cell lines (Bogenmann et al., 1988).

In vivo topological studies show that retinoblastoma evolution is radially asymmetrical and mimics the patterning and distribution of red/green cones (Munier et al., 1994). Naturally, cone precursor cells express unusually high levels of pRB during maturation alongside oncoproteins MDM2 and MYCN. These proteins are essential for the proliferation and survival of the cell (Xu et al., 2009). Evidence suggests that the cone precursor cell is indeed the Rb cell of origin.

Indeed, it was observed that *Rb1* depletion in cone precursor cells derived from foetal retinal tissue and transplantation into orthotopic xenografts generates tumours suggesting that cone precursor cells are the cell of origin. However, the tumours that formed had different histology to retinoblastomas and did not harbour significant DNA alternations (Xu et al., 2014).

It has been a challenge to demonstrate Rb development and evolution in vivo due to the lack of appropriate disease models. No known naturally occurring animal models exist for this distinctively human disease. The generation of transgenic mice has been important in the pre‐clinical testing of novel therapeutics against Rb but is limited as mutations in *Rb1* do not form retinoblastomas. Instead, other proteins of the Rb pathway need to be disrupted to impose tumorigenesis. Even still, these models remain phenotypically distinct from human Rb with amacrine and horizontal cell involvement instead of cone precursor cells (Mendel & Daniels, 2019).

### 4.6 Disease modelling with iPSC‐derived tissues

As Rb is a disease of the developing retina, it is a perfect disease candidate to model with PSC‐derived ROs. Several groups, including our own, have utilised this technology to uncover missing links in the pathogenesis of retinoblastoma. Previously, in ROs derived from RB1‐null hESCs, it was observed that pRB expression is enriched in RPCs and that loss of RB1 promotes cell cycle entry. Significantly fewer viable PRs were present as well as reduced bipolar and ganglion cells. However, no evidence of tumorigenesis was observed in vitro or in vivo in immunocompromised mice suggesting while pRB is crucial for retinogenesis, loss of its function is not sufficient alone for tumorigenesis (Zheng et al., 2020).

Interestingly, in another study ROs derived from hESCs with biallelic mutation (p.R320X) in *Rb1*, displayed evidence of tumorigenesis in vitro, comparable with in vivo Rb features such as transcriptome and genome‐wide methylation and upregulation of SYK pathway. They also report the cell of origin to be Arrestin‐3^+^ cone precursor cells (Liu et al., 2020). However, this study negated the influence of cell‐line‐specific genetic background for Rb generation with the use of one cell line.

The influence of patient background on RB formation was assessed in one such study where 15 participants with germline *Rb1* mutations were reprogrammed to iPSC and differentiated to 3D ROs using an optimised 3D retinal organoid protocol. ROs from each patient were matured for 45 days and at which point were dissociated and injected intravitreally into immunocompromised mice to investigate in vivo tumorigenesis. As expected, each mouse developed retinal tumours, distinct from teratomas. Whole‐genome sequencing revealed inactivation in the second allele of the *RB1* gene with no other cancer gene aberrations (Norrie et al., 2021). Copy number gains were observed in *MDM4* and *MYCN*, a feature commonly observed in retinoblastomas (Thériault et al., 2014). Further to this, RNA sequencing demonstrated a high degree of similarity between the iPSC‐RBs and human retinoblastomas. Epigenetic dysregulation was also evident with the upregulation of SYK RNA and protein, a known oncogene necessary for tumorigenesis in retinoblastoma.

Through single‐cell RNA sequencing (scRNA‐seq), RPCs were the most common cell identified in a pool of RB tissues including orthotopic patient‐derived xenografts, healthy adult retinas, retinoblastomas and iPSC‐RBs, corresponding to 53% of the cells in this subset. This was closely followed by rod photoreceptors making up 31% of the total cells sequenced. An interesting observation they note is the hybrid gene expression phenotype displayed by individual RB tumour cells which are not present in normal retinogenesis. Their study suggests that the cell of origin is RPCs, and the hybrid gene expression observed can be explained by their multipotency in retinal development (Norrie et al., 2021). This formative paper demonstrated the power and reproducibility of Rb modelling with PSC‐derived ROs and provided valuable information that could be useful in the prognosis of paediatric cancers.

### 4.7 Shifting the focus towards transcriptional events during retinogenesis

Many studies to date have focused on the end‐stage of tumour development in Rb. However, our research group is particularly interested in the transcriptional events taking place during cell state transitions that preclude Rb development (Rozanska et al., 2022). To explore this, an RB1‐null hESC line was genetically engineered with CRISPR/Cas9 technology, alongside the reprogramming of somatic cells from a patient carrying a heterozygous germline *RB1* mutation (c.2082delC). The mutational status of these cell lines, alongside isogenic controls, allows for a thorough molecular investigation of Rb development and for the acquisition of novel insights regarding in vitro tumorigenesis.

**FIGURE 6:**
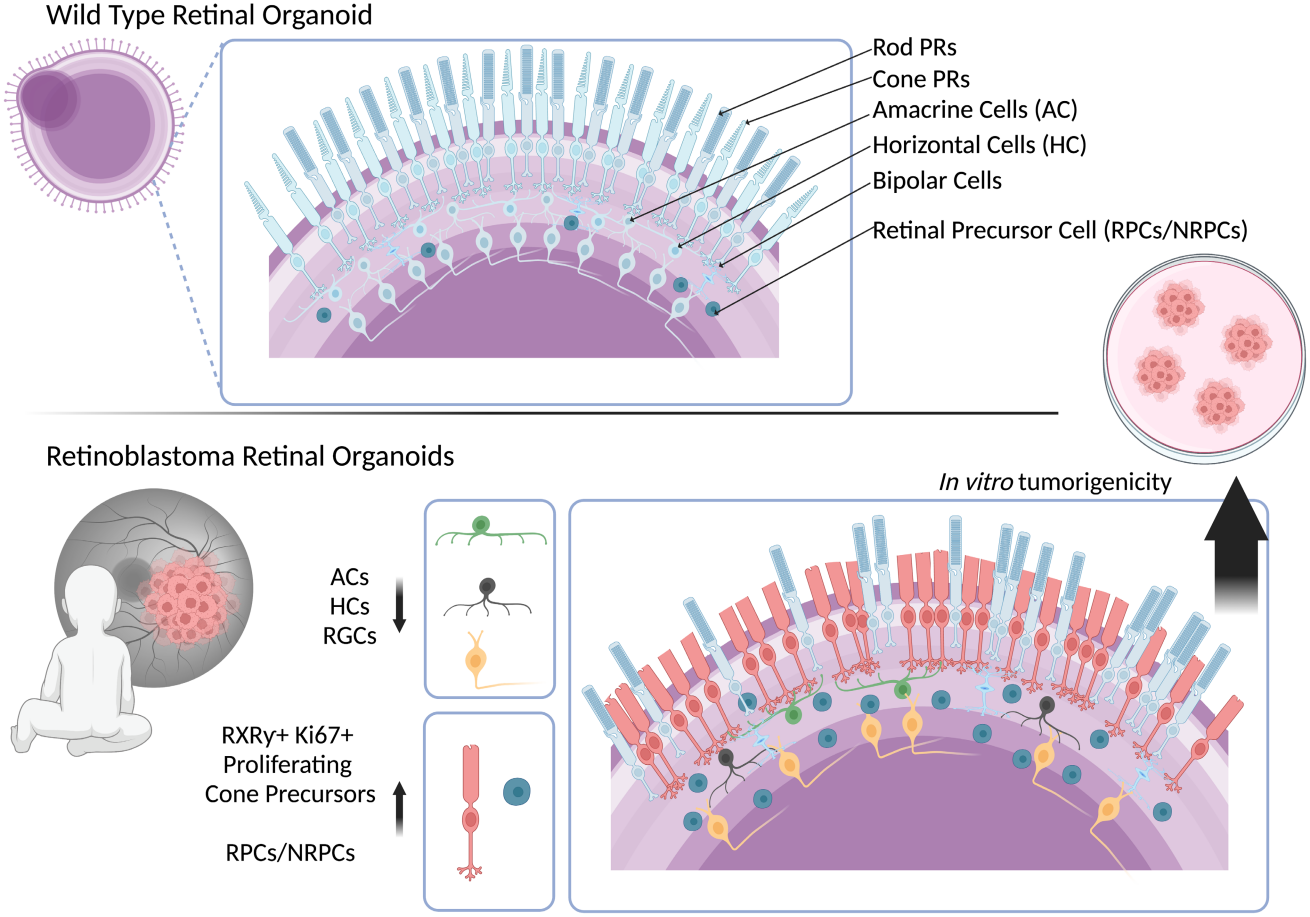
Retinoblastoma phenotype in retinal organoids. Retinal organoids recapitulate retinogenesis and contain all key retinal neurons including rod photoreceptors, cone photoreceptors, amacrine cells (ACs), horizontal cells (HCs), bipolar cells, retinal precursor cells (RPCs/NRPCs) and retinal ganglion cells (RGCs). This enables cell‐specific phenotyping in disease models. Retinoblastoma, a childhood cancer, has been shown to delay development corresponding retinal organoids with an increase in retinal progenitor cells. An accumulation of cone photoreceptor precursor cells suggests they are the cell of origin for Retinoblastoma. Deficiencies in amacrine cells, horizontal cells, and retinal ganglion cells are also observed in a disease‐specific manner. Figure adapted from Rozanska *et al*. (2022).

One key phenotype observed in our models was the significant decrease in amacrine cells observed alongside the increase in RXRƴ^+^ Ki67^+^ cone precursor cells, and retinal progenitors in all RB1‐ROs—see Figure 6. Active caspase‐3 was also significantly upregulated in the retinal organoids and corroborates the increased rates of apoptosis seen in mouse models (Zheng et al., 2020). Through scRNA‐seq analysis, we hypothesize that the accumulation of RPCs in RB1‐deficient ROs is a result of a block in differentiation due to the lack of pRB, which is required for exit from the cell cycle. This occurs either before or after the emergence of transient neurogenic RPCs in our organoids and corroborates findings seen in previous studies (Chen et al., 1996; Zhang et al., 2004).

The cellular transformation was evident from the culturing of dissociated cells of RB1‐null ROs in soft agar, a gold standard assay for assessing the tumorigenic potential of a cell (Au—Borowicz et al., 2014). This was deduced by the appearance of small and large aggregates that had formed in the agar in an anchorage‐independent manner. In support of this, 200‐day‐old RB1‐null ROs displayed tumorigenic features in histological analysis with the formation of neural rosettes enriched in progenitor (VSX2) and proliferative markers (Ki67) and comparable to Flexner‐Wintersteiner and Homer Wright rosettes, a common pathological feature of Rb (Wippold & Perry, 2006).

A novel aspect of this study was that RB1‐null ROs were cultured for long periods of up to 150 days, enabling an apt time frame for the appearance of tumorigenic properties in vitro. Such a model is not only invaluable for understanding the molecular pathogenesis of RB1 but also in the drug screening and toxicology profiling of candidate drugs for pre‐clinical trials.

As a proof of principle drug screening experiment, 150‐day‐old RB‐null ROs were incubated with three different drugs commonplace in the treatment of RB: Melphalan, Topotecan and TW‐37 for 72 h. Quantitative immunofluorescence of resulting ROs revealed a significant decrease in the quantity of proliferating cone precursors at specific doses for each drug, validating the RB1‐ROs as a robust platform for drug screening.

## 5 CONCLUSION

iPSC‐derived retinal models have proven to be invaluable in the study of retinal diseases. This tool specifically gives insight into a window of development that precludes the onset of disease in humans. This time frame is often disregarded in the study of pathogeneses of retinal disease as disease phenotypes often present postnatally. With retinal disease, it is important to treat affected individuals before the onset of neuronal cell death as neurons are post‐mitotic and cannot regenerate. In that regard, it is important to interrogate the potential influence of mutations in retinal disease genes during the process of retinogenesis. There are likely clues hidden in the molecular signatures of these diseases during development which could provide novel disease biomarkers for prenatal screening or novel therapeutic avenues to explore, providing opportunities to prevent and treat the retinal disease before a loss of vision.

This review provides ample evidence of the validity of retinal organoids for the study of retinogenesis as their ability to recapitulate many features of in vivo retinal disease. ROs can provide endless opportunities to study disease from a patient‐specific context, which is often not possible with animal models. However, like any model, ROs also present some limitations to their use. Retinal development continues postnatally. ROs do not offer this view of retinal disease, particularly in diseases that are age‐related—which need additional stressors to recapitulate in vivo phenotypes. ROs will likely not replace in vivo animal models completely but instead, serve as a tool to be used in conjunction with in vivo or in silico modelling of retinal disease. Further to this, many diseases involve both neural retina and RPE, but existing RO models are not able to produce anatomically correct and functional RPE in situ. This affects the maturation of photoreceptor outer segments and ultimately their functional capacity in terms of the light response. Future work is focused on developing co‐cultured models of ROs and RPE for the purpose of transplantation and more accurate disease modelling.

## Data Availability

Data sharing is not applicable to this article as no new data were created or analyzed in this study.
